# Role of lipid transfer proteins in loading CD1 antigen-presenting molecules

**DOI:** 10.1194/jlr.R083212

**Published:** 2018-03-19

**Authors:** Luc Teyton

**Affiliations:** Department of Immunology and Microbiology, Scripps Research Institute, La Jolla, CA 92037

**Keywords:** endosome, lipid exchange, lipidome

## Abstract

Research to connect lipids with immunology is growing, but details about the specific roles of lipid transfer proteins (LTPs) in antigen presentation remain unclear. A single class of major histocompatibility class-like molecules, called CD1 molecules, can present lipids and glycolipids to the immune system. These molecules all have a common hydrophobic antigen-binding groove. The loading of this groove with various lipids throughout the life of a CD1 molecule defines the immune recognition of lipids by T cells. At each location of residence, CD1 molecules are exposed to particular physicochemical conditions, particular collections of lipids, and unique combinations of LTPs that will define which lipids bind to CD1 and which do not. The lipid transfer machinery that is used by CD1 molecules is entirely hijacked from the normal synthetic and catalytic pathways of lipids. The precise determinants that regulate the presentation of certain lipids over others with respect to chemistry, solubility, and abundance are still poorly defined and require investigation to allow the use of lipids as regular antigenic targets of immunotherapy and vaccine.

Lipids were mostly ignored by immunologists for the longest time. Not considered immunogenic or antigenic in most systems, the only place for lipids was inflammation, and inflammation was barely part of immunology. Beyond decades of work on prostaglandins and leukotrienes, the slow emergence of the molecular recognition of lipopolysaccharides (LPSs) brought inflammation slowly back into the resurgent field of innate immunity. The binding of LPS to LPS binding protein (LBP) (1) and CD14 after transfer from LBP (2) established two principles: immune receptors were sensing microbial lipids and molecular transfer reactions were necessary for translating extracellular information to cells. This line of research culminated in the identification of the cellular signaling receptor of LPS, Toll-like receptor 4 (3). At about the same time, a new family of major histocompatibility complex (MHC)-like genes, called CD1, had been identified by cloning (4) and shown to encode molecules that could stimulate T cells (5). A year later, still in search of its function, the mouse CD1d molecule was crystallized and its structure solved by Wilson and colleagues (6), who noted an unusual large hydrophobic groove for this MHC-like molecule, departing clearly from other MHC molecules. A series of reports confirmed that, indeed, lipids and glycolipids were presented by human CD1 molecules (7–9). For the unique murine CD1d molecule, serendipity unlocked the puzzle when a glycolipid isolated from a marine sponge was discovered as being a ligand for a small unusual population of T cells, termed invariant natural killer T (NKT) cells, and restricted by CD1d molecules (10). Since then, many endogenous and exogenous lipids and glycolipids have been identified as being recognized by αβ T cells and γδ T cells in the context of the CD1 family members A, B, C, and D for humans and some other species, and D in the mouse (11). Despite these important discoveries, the study of certain aspects of the immune recognition of lipids have been limited due to inherent technical issues: *1*) CD1 molecules are integral membrane proteins and their biochemical interrogation necessitates the use of detergents; *2*) the use of detergents, depending on their properties, will alter the lipid composition of CD1-associated lipids; and *3*) labeling and detection of lipids and glycolipids are not as sensitive and easy to use as the analytical techniques available to protein biochemists. Because of these hindrances, studies looking at the cell biology and antigen presentation of lipids onto CD1 molecules are scarce, even if some of the most pressing issues have been at least partially answered: assembly of CD1 molecules in the endoplasmic reticulum (ER) (12); trafficking to the cell surface (13); cellular location of loading (13); uptake of exogenous lipids (14); and identification of endogenous ligands (15). In addition, all mutations pertaining to lipid antigen processing and loading in human and animal models are dominated by neurological issues that are so prevalent that immune phenotypes have rarely been studied in their context. This situation highlights the fact that to handle lipids, the immune system has simply hijacked a molecular machinery that was already existing and critical for some of the functions of lipids in organs such as the central nervous system, as well as metabolism.

## CD1 MOLECULES

CD1 molecules are non-MHC-encoded MHC-like molecules that share structural features with MHC class I, such as the association with β2 microglobulin, and functional features of MHC class II molecules, such as the possible association and trafficking with the invariant chain (16). Their main difference from other MHC and MHC-like molecules is the nature of the groove where the antigen will be displayed: it is made of hydrophobic residues that can only accommodate lipid chains or very hydrophobic side chains of amino acids (6). This groove is made of two large pockets, A’ and F’, with the exception of CD1b in humans, which is expanded by a smaller C’ pocket between A’ and F’ and a T’ tunnel to the front of the C’ pocket (17). The A’ pocket is always fully occupied by a single lipid chain of up to C26 in length, or by a shorter lipid chain and a spacer fatty acid (18), while the F’ pocket is more permissive, allowing short lipid chains partial occupancy as well as longer lipids protruding out, exposing them to T cell recognition (19). The nature of the lipids that anchor in the lipid groove of CD1 molecules covers most known lipid structures: phospholipids, fatty acids, glycolipids, glycerolipids, lysolipids, tetraacylated lipids, and lipopeptides; and an unlimited variety of head groups: single monosaccharides, disaccharides, trisaccharides, tetrasaccharides, and peptides (19). Beyond this long list, with respect to loading to CD1 molecules, the apparent ability of most, if not all, lipids to be able to bind questions the way a hierarchy is established for immune presentation. Indeed, purely based on abundance and critical micelle concentration (cmc), lipids such as phosphatidylcholine and lysolipids would always be over-represented, while rare microbial lipids might not be displayed in high enough quantities to trigger immune responses. This important question has not been resolved by a limited number of studies that have examined the natural lipidome bound to secreted or cell surface-cleavable CD1 molecules at steady state (20, 21). In those studies, the most abundant cellular lipids were found in association with CD1. Of course, the experimental setup needed to purify CD1 molecules and examine their lipid content greatly limits the conclusions that one might draw from these results, but a technique that would allow the interrogation of CD1-bound lipids to membrane-anchored molecules in dynamic situations, e.g., infection, is not available yet. In any case, similar to the loading of peptides onto classical MHC molecules, a few clever pathways have emerged in evolution to allow important lipids to be displayed on CD1 molecules in critical condition; they will be discussed below.

## PROCESSING AND LOADING: LOCATION

**Fig. 1.**
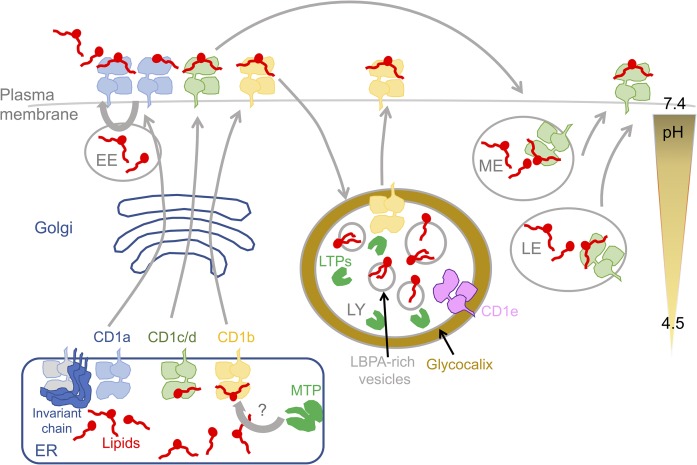
Schematic representation of the biology of lipid presentation by CD1 molecules. EE, early endosome; ME, medial endosome; LE, late endosome; LY, lysosome.

With the exception of phosphatidylglycerol and cardiolipin, which are synthesized in and confined to mitochondria, glycerolipids and sphingolipids are synthesized in the ER and the cytoplasm, and traffic as an integral part of membranes to be modified and/or reach their final location or as monomers in association with lipid transfer proteins (LTPs), such as CERT, FAPP2 (22), or ganglioside GLTP (23) in the case of sphingolipids. Catabolism of lipids occurs in most cellular compartments, from the cell surface where phospholipases actively modify the cell membrane, the cytoplasm with a number of neutral sphingomyelinases and lipases, the ER with neutral lipid hydrolases, and the entire endosomal pathway from early endosome to lysosome where a series of acid sensitive hydrolases will be activated progressively as their concentration increases and the pH drops. With respect to lipid biology, the lysosome has two essential complementary functions: first, it has a pivotal role in metabolic sensing and will produce fatty acids as a source of energy in stress conditions (24); second, it controls the catabolism of most essential glycolipids, especially ceramides (25), a requirement for the survival of cells under stress. For loading, CD1 molecules are found mainly in three locations: the ER, the cell surface, and the endocytic compartments. These exclusive locations certainly limit the number of lipids that CD1 molecules can bind, as each is associated to unique physical conditions that influence binding (**Fig. 1**). In addition, as we had learned from classical peptide-binding MHC molecules, ligands do not load themselves into the groove but require molecular assistance, tapasin for MHC class I (26) and DM for MHC class II (27). It was likely that the same principle applied to CD1 molecules, especially given the fact that lipids are rarely found free in solution at concentrations below their cmc, but are usually associated with membranes or bound to LTPs. While some of these LTPs are mere transporters, like the albumin family, some, the LTPs, are endowed with transfer capacities, each linked to unique functions and often unique subcellular locations (**Table 1**). Therefore, the distribution of LTPs and their local coincidence with CD1 molecules will ultimately determine which lipid is loaded onto the immune receptor. Finally, it is important to mention that studying the cell biology of lipid loading onto CD1 molecules remains extremely difficult; while the detection of the protein moiety is accessible to a number of antibodies, the direct detection of the lipid and the specification of its nature are very challenging. Only a few antibodies can see lipids or glycolipids (15) or complexes between CD1 and lipids (28). These difficulties account largely for some of the discrepancies that have been seen between laboratories or simply between mouse and human (29), as the range of reagents varies greatly.

**TABLE 1.** Respective role of some LTPs in lipid metabolism and CD1 antigen loading

Name of LTP	Role in Lipid Metabolism	Role in CD1 Antigen Presentation
MTP	ER assembly of apoB	ER loading of lipids
NPC1	Cholesterol transport	Important for the traffic of CD1d ligands
NPC2	Cholesterol transport	Loading of glycolipids into CD1d
GM2A	Molecular chaperone for β-hexosaminidase A	Unknown
GLTP	Cytoplasmic trafficking of sphingolipids	Unknown
Prosaposin	Unknown function before cleavage	Unknown
SapA	Molecular chaperone for GALC	No direct CD1-unique lipid relationship described yet
SapB	Molecular chaperone for GLA	Essential for loading endogenous ligands onto CD1d
SapC	Molecular chaperone for GBA	Important for loading many lipids onto CD1b
		Necessary for the loading of cardiolipin onto CD1d
SapD	Molecular chaperone for ASAH1	No direct CD1-unique lipid relationship described yet
CD1e	Unknown	Important for loading PIMs onto CD1b

GALC, galactocerebrosidase; PIM, phosphatidyl-myo-inositol.

## THE ER

The ER is a very active, synthetic, and catalytic compartment for lipids and the main reserve of membranes in a cell. The ER produces the essential phospholipids that are assembled to produce membranes and can do so in stress conditions through the unfolded protein response (30), a pathway that is induced during immune responses (31). The ER is where CD1 molecules are assembled before export through the Golgi, following a succession of interactions with chaperones that is very similar to what has been described for MHC class I molecules (12). It is known for MHC class I that the release from the ER chaperone complex is peptide dependent, while for MHC class II it is linked to the binding to the invariant chain (32). Thus, what allows the release of CD1 molecules? In part, the invariant chain carries a similar function for CD1 as it does for MHC class II (16, 33), but it appears that only a fraction of CD1 molecules is associated with the invariant chain. So, the question of what lipids might be bound to CD1 heterodimers when they leave the ER is still open. One important observation has been made with respect to potential interactions with ER LTPs: the microsomal triglyceride transfer protein (MTP) normally responsible for the lipidation of apoB has been shown to associate with CD1d and influence its functions in hepatocytes (34, 35). However, the precise mechanisms by which MTP modifies the biology of CD1 molecules remains unclear in the absence of biochemical methods that would give direct access to the composition of the CD1 lipidome in the presence and absence of MTP. One can assume, based on what classical MHC molecules do, that the CD1 groove is never empty and would unfold in the absence of a ligand. However, because it is so hydrophobic, the CD1 groove could collapse on itself in the absence of a bound lipid and prevent denaturation of the whole molecule (36); there is currently no experimental data to support this observation.

## TRANSPORT THROUGH THE GOLGI APPARATUS AND ACCESS TO THE CELL SURFACE

There is also no evidence to indicate that this potential lipid cargo is altered during the transit of CD1 molecules through the Golgi apparatus en route to the cell surface, but no study has addressed the issue. Once at the cell surface, it is known that lipids with low cmc can readily exchange CD1-bound lipids and be presented to immune cells (18). In the particular case of NKT cell agonists, this possibility has been used to stimulate cells without having to use the normal transport and endosomal uptake for presentation in order to produce unique functional outcomes (37, 38). In such a way, short fatty acid variants of α galactosylceramide are very soluble and can load directly at the surface of antigen-presenting cells, and have very short half-lives that result in T_H2_-biased immune responses (18, 37) and benefit some T_H1_-mediated autoimmune conditions (37, 39)_._ It is not known to what extent soluble extracellular lipids, such as short fatty acids and extracellular LTPs, can exchange CD1-bound lipids when CD1 is expressed at the cell surface. What is known is that with the exception of CD1a, all human and murine CD1 molecules will get internalized by using the association of their cytoplasmic tails with AP2 and AP3 to reach the early and late endosomal compartments, respectively (40). CD1a, absent in mice and rats, appears to behave like cell surface molecules and readily exchange lipids without internalization or by simply accessing the early endosomes where pH is above 6.5 and acid-activated LTPs are in low abundance and mostly inactive (41).

## THE ENDOSOMAL PATHWAY AND THE LYSOSOME

### Antigen processing

Like MHC class II molecules, the endosomal pathway is where CD1 molecules will acquire most antigens relevant to their presentation functions. This pathway that ends with the lysosome and is essential in the production of cellular nutrients is also the location of antigen processing and loading. With respect to processing, the endosome has the dual role of processing not just the antigens but the processing enzymes themselves; this latter necessary function is accomplished by pH-induced self-catalysis and/or by protease cleavage and is likely to be important for the presentation of certain lipids. As the best illustration of this phenomenon, cathepsin L deficiency has been shown to have a profound effect on the selection of NKT cells and the production of their endogenous selecting ligands (42). It is also known that some glycolipids require processing of their head group for presentation to T cells, but very few examples have been documented. For instance, the artificial Gal(α1-2)GalCer glycolipid needs to be processed by acid α galactosidase (GLA) to be recognized by NKT cells (43), and this enzyme requires the assistance of the small lysosomal LTP, saposin (Sap)B, for activity (44). Similarly, the mycobacterial hexamannosylated phosphatidyl-myo-inositol requires processing by acid α mannosidase to be recognized in the context of CD1b by T cells (45). In addition, these same antigens require the activity of lipases to modify the lengths of the acyl chains and make them compatible with the CD1b groove; this trimming is accomplished by pancreatic lipase-related protein 2 (PLRP2) and lysosomal phospholipase A2 (LPLA2) (46).

### Antigen loading

For many years, the search for a molecule capable of assisting the loading of lipids onto CD1 molecules was focused on the concept that had developed for MHC class II and its enzymatic editor, DM. The human genome, in addition to its four isotypes, retains a fifth CD1 molecule, CD1e, which quickly drew parallels with HLA-DM because no lipid binding could be demonstrated for this fifth member of the family. The work of de la Salle and colleagues on CD1e demonstrated that, for some glycolipids, CD1e could assist in the processing (45) and most likely also allow the exchange of some lipids from CD1b (47). However, it does not appear that CD1e is necessary for many other lipids, as natural variants of CD1e have only very limited deficiencies in their ability to present glycolipids (48). In addition, CD1e is absent from species that express only CD1D molecules, such as the mouse or the rat. So why is there such a narrow specificity? A selection event based on a unique infectious agent could be the explanation.

Other groups, including our own, focused their search for molecular assistants of lipid loading on the small group of known LTPs that reside in the endosomal pathway, especially the lysosome. These molecules are ganglioside monosialic 2 activator (GM2A), Niemann-Pick type C (NPC)2, SapA, SapB, SapC, and SapD. All are activated at acidic pH, although optimal pH varies for each. Structurally speaking, they split into two families, ML that includes GM2A and NPC2 as well as MD-1 and MD-2, and SAPLIP with the four Saps and a number of disparate proteins such as surfactant protein B, NK lysin, granulysin, AOAH, and sphingomyelinase (49). The ML family members are about 150 amino acids long and folded around four conserved cysteines that create a central hydrophobic cavity in which acyl chains are bound. The SAPLIP proteins are smaller, at around 80 amino acids in length with six conserved cysteines, and accommodate lipid chains at the interface they form when they multimerize at acidic pH (50, 51). The precise distribution of these LTPs in each of the discrete endocytic compartments is unknown, as very few antibodies specific for each have been produced. The four Saps have a common precursor, prosaposin, which is secreted and internalized by LRP1, sortilin, and the mannose-6-phosphate receptor (52), and cleaved as pH decreases throughout the endocytic route. The ability of prosaposin and the processing intermediates (tri- and disaposin) to transfer lipids has been suggested in various studies, but has never been formally demonstrated in vivo. As already mentioned, each of these small LTPs has a fundamental role in assisting catabolic enzymes of the sphingolipid and glycolipid pathways to carry out their hydrolytic functions. As such, their deficiencies in animals and humans are marked by profound neurological phenotypes (53). Prior to examining immunological phenotypes in knockout animals, we tested the concept that lysosomal LTPs could transfer lipids and glycolipids onto recombinant mouse and human CD1 molecules in vitro. All six lysosomal LTPs, as well as the cytoplasmic GLTP (23), were recombinantly expressed and tested in an in vitro assay for their ability to load sulfatide and α galactosylceramide (54). Surprisingly, all seven molecules were capable of various degrees of transfer, establishing the principle of lipid exchange onto the CD1 molecule, but not investigating its biological relevance. Because of its cytoplasmic location, it was obvious that GLTP was not involved in CD1 loading in vivo. The other six molecules were tested in knockout animal models. No phenotype could be associated with GM2A deficiency with respect to lipid presentation and/or selection of lipid-specific T cells, such as NKT cells, even though GM2A transfers α galactosylceramide very efficiently in vitro (L. Teyton, unpublished observations). On the other hand, the phenotype in prosaposin knockout animals was dramatic with a complete loss of the presentation of endogenous selecting ligands for NKT cells and an inability to present exogenous antigens (55). The same phenotype was present in SapB knockout animals (L. Teyton, unpublished observations) and in NPC2-deficient animals (56). While NPC2 can load a variety of glycolipids into CD1 molecules and have a direct effect on their biology, it is also essential for the efflux of cholesterol from the lysosome, together with NPC1. Not surprisingly, cholesterol has an essential role in the anatomy and dynamics of endocytic vesicles and indirectly on the function of all LTPs and CD1 biology. The knockout of NPC1 profoundly alters the trafficking of NKT cell ligands and CD1 molecules (57). This traffic of cholesterol is primordial for the formation of bis(monoacylglycero)phosphate (BMP)- or LBPA-rich vesicles and multivesicular bodies (58), as BMP enrichment is paired with cholesterol depletion and the sprouting of small high-curvature vesicles that expose lipids and glycolipids to the extraction by LTPs (59) (Fig. 1). The mechanisms of extraction of lipids from membranes by LTPs are reasonably well-understood, especially for GM2A (60), but the transfer to soluble or membrane-bound hydrolases or CD1 molecules remains unclear. All LTPs can transfer lipids between liposomes, but the molecular determinants that control binding versus release are unknown. In the case of GM2A, direct interaction with hexoaminidase A occurs, but it does not appear to influence lipid binding (60). We have not been able to reliably measure an interaction between any mouse or human CD1 molecules and the four Saps, indicating that if this interaction happens, it is weak, very transitory, and unlikely to provide specificity between any Sap and any CD1 molecules. However, it has been suggested that lipid editing on human CD1b is more sensitive to SapC (61, 62); this leaves open the possibility that some Saps have a degree of specificity, but it is more likely that SapC is preferred by CD1b because its functional trimeric nature endows it with the capacity of binding larger lipids that preferentially bind to CD1b (50, 63). In all cases, Saps are known to bind a large collection of lipids and have important overlap of specificity within each group of them. Our studies and those of others (64, 65) have focused attention mainly on SapB, but the role of the other three Saps and their potential cooperativity have not been systematically addressed. The association of particular Saps with the enzymes that are important for the catabolism of many CD1 ligands is better understood. For instance, the degradation of the natural endogenous thymic ligands of NKT cells, α galactosylceramide and α glucosylceramide, requires, first, the action of acid ceramidase (ASAH1) to remove the fatty acid before the GLA and acid α glucosidase (GAA), respectively, can remove the head glycan (15). While ASAH1 is dependent on SapD (66) for activity, GLA requires SapB (67). No Sap dependency has been described for GAA, but acid β galactosidase (GBA) that cleaves β-glucose is entirely dependent on SapC for activity (68). The requirement for LTPs for both loading and degradation of CD1 ligands makes them the rheostat of lipid presentation by CD1 receptors. Therefore, lipid presentation will be the complex result of the presence of a particular set of lipids in an endosomal vesicle, the concentration of cholesterol and BMP, the isotype and abundance of CD1 molecules, the mixture and respective abundance of the six lysosomal LTPs, and the pH of this compartment.

## CELL SURFACE

Once expressed at the cell surface of antigen-presenting cells, CD1-lipid complexes are not only displayed for T cell recognition but also exposed to the LTP-rich extracellular medium. If it is known that many lipoproteins, such as apoE, can transport immunogenic lipids (14, 69), the role that lipoproteins may play in the possible unloading of cell surface CD1 molecules has not been studied yet. It would be of great interest to examine the role of LBP, CD14, and AOAH, as all are important molecules in the innate phase of immunity. For instance, we have shown in vitro that LBP could help with the loading of lipids into CD1d (L. Teyton, unpublished observations) and AOAH retains a Sap-like domain. Lipid exchange activities at the cell surface could be essential for the half-life of all CD1 molecules.

## CONCLUSIONS

The recognition of lipids is central to homeostatic functions and anti-microbial responses of the immune system. This role is attributed to two families of molecules with little in common: the CD1 molecules, which have evolved from classical MHC genes by duplication and selection, and the lysosomal LTPs, which were evolved to assist catabolic enzymes to hydrolyze lipids. The overlapping specificity of binding of each family of molecules creates a landscape of lipid antigen presentation that we still do not understand fully and that remains difficult to study using the classical biochemical tools that we have at hand. Ultimately, our ability to deconstruct this system might allow immunologists to design vaccines in which lipids will be the antigenic determinants.
